# Bringing the Animal QTLdb and CorrDB into the future: meeting new challenges and providing updated services

**DOI:** 10.1093/nar/gkab1116

**Published:** 2021-11-24

**Authors:** Zhi-Liang Hu, Carissa A Park, James M Reecy

**Affiliations:** Department of Animal Science, Iowa State University, 2255 Kildee Hall, Ames, IA 50011, USA; Department of Animal Science, Iowa State University, 2255 Kildee Hall, Ames, IA 50011, USA; Department of Animal Science, Iowa State University, 2255 Kildee Hall, Ames, IA 50011, USA

## Abstract

The Animal QTLdb (https://www.animalgenome.org/QTLdb) and CorrDB (https://www.animalgenome.org/CorrDB) are unique resources for livestock animal genetics and genomics research which have been used extensively by the international livestock genome research community. This is largely due to the active development of the databases over the years to keep up with the rapid advancement of genome sciences. The ongoing development has ensured that these databases provide researchers not only with continually updated data but also with new web tools to disseminate the data. Through our continued efforts, the databases have evolved from the original Pig QTLdb for cross-experiment QTL data comparisons to an Animal QTLdb hosting 220 401 QTL, SNP association and eQTL data linking phenotype to genotype for 2210 traits. In addition, there are 23 552 correlations for 866 traits and 4273 heritability data on 1069 traits in CorrDB. All these data were curated from 3157 publications that cover seven livestock species. Along with the continued data curation, new species, additional genome builds, and new functions and features have been built into the databases as well. Standardized procedures to support data mapping on multiple species/genome builds and the ability to browse data based on linked ontology terms are highlights of the recent developments.

## INTRODUCTION

**Figure 1.**
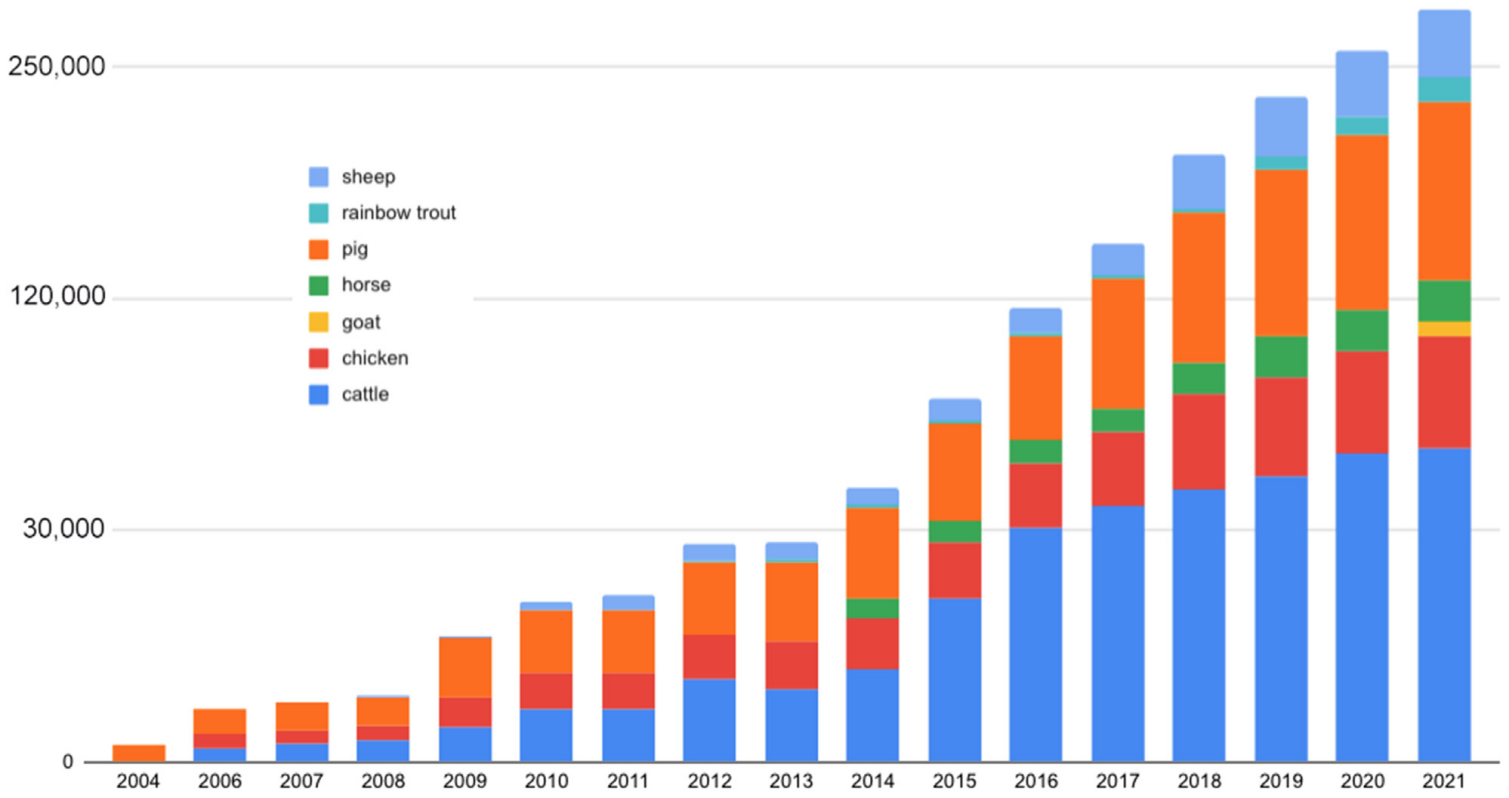
Cumulative curated data increase in all seven species hosted at the Animal QTLdb/CorrDB (according to database release records; data transformation was performed on data counts in order for small counts to be visible).

Over the past two decades, livestock genomics research has been greatly accelerated by advances in high-throughput genomics technologies. Not only are whole-genome assemblies available for every major livestock species (1), but multiple genome builds within each species for major livestock breeds have been or are being developed (2). In the meantime, continued discovery of single nucleotide polymorphisms (SNPs) has enabled the generation of high-throughput genotype information from different SNP chip platforms and production of massive genotype–phenotype data from association studies, trait correlation analysis, and heritability estimates (3). This is reflected in the steady data increase in the QTLdb/CorrDB in all seven livestock species (Figure 1).

Although efforts to functionally annotate gene expression information are progressing (4), addressing the need for rapid access to and interpretation of all the associated data remains an increasingly challenging task.

**Figure 2.**
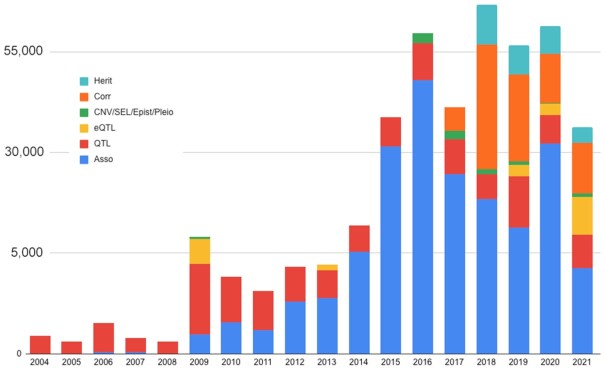
A stacked column plot showing a significant increase of curated data by data types in recent years (Notes: (i) the data for 2021 are mid-year counts; (ii) counts for all data types are transformed for small values to be visible; (iii) prior to 2009, the database did not have a full-time curator).

As the Animal QTLdb has been modified over time to accept curation of multiple data types (e.g. SNP associations, eQTL, copy number variations (CNV), signatures of selection, epistasis, pleiotropy), and curation of correlation and heritability data into the CorrDB has continued (5), the curation workload has increased significantly (Figure 2). We have been able to boost curation efficiency by developing new tools and structures to improve the dataflow with built-in quality controls. The Animal QTLdb and CorrDB have been developed in tandem to minimize duplication of effort. Both databases have undergone three data releases per year since our last report (6). In this paper, we present our ongoing efforts to further develop the QTLdb and CorrDB with a more elastic infrastructure to increase flexibility for inclusion of new types of data entries, new genome builds for genome map data lift-over, new ontology tools for linking information, and additional tools to assist users with viewing and analyzing data.

There are five essential data entities in the QTLdb/CorrDB: trait information, map information (QTLdb only), gene information (for eQTL data only), animal information, and publication/experiment information. The major improvements on tools and procedures we have made to facilitate the curation of two of these, trait and map information, will make these databases better positioned to meet upcoming needs.

## NEW DEVELOPMENTS

### Database structure improvements and standardized protocol for adding new species to QTLdb/CorrDB

The QTLdb was initially developed for a single species (7). Other species were added by using a copy of the database and tool set, then manually adding the necessary parts, such as set of traits, genome/linkage maps, list of markers on these maps, etc., before any data could be curated for that species. To streamline this process, we have developed a standardized protocol for adding a new species which is easily operable as a routine. For this purpose, a set of linked web forms was set up to administer the introduction of a new species, initiate trait entry for the new species (Supplementary data, Figures S1–S3), and introduce a new genome map/assembly (detailed below). The set of web forms serves not only to simplify information input but more importantly to enforce the entry of required information in order to main database integrity, and functions as an information checkpoint (Supplementary data, Figure S4). This pipeline was tested during the recent addition of goat (a new species) and a new genome build for an existing species (sheep) to the QTLdb/CorrDB.

### Livestock SNP ID/name matching repository to assist SNP-based data curation

One of the requirements for entering SNP association data into the Animal QTLdb is that a map location must be represented using a permanent official SNP ID, or ‘rs number’, to ensure data persistence. This means that the SNPs used in experiments/reports must be submitted to, processed, and released by a public SNP archive (non-human organism SNP data was previously hosted at NCBI, https://www.ncbi.nlm.nih.gov/snp/, and is currently at the EMBL-EBI European Variation Archive [EVA], https://www.ebi.ac.uk/eva/). However, due to the exponential increase of SNP data and limited resources to process them, the current release cycle at the EVA can cause very long delays at Animal QTLdb as we wait for the official IDs to become available, creating a bottleneck in our release of curated data. This temporary unavailability of official SNP ‘rs numbers’ interferes with timely data curation and release, because the SNP-based QTL/association experiment is carried out and results published ahead of the EVA release of the SNPs used. Often custom SNP identifiers, rather than universally stable ‘rs’ IDs, are used in QTL/association data publications.

Custom SNP IDs/names usually come from SNP discovery workflows, genotyping platforms, and data integration and comparison processes. They may appear in scientific reports prior to SNP data being submitted to or released by a public repository. Not only are there long intervals between submission of SNP data and assignation of stable ‘rs’ IDs, but the original custom SNP identifiers are often not linked to the final SNP IDs. The SNP identity matches became an essential part of our curation of SNP-based QTL/association data.

To overcome these difficulties, we reached out to the research community to collect original or secondary data that provide the custom SNP IDs/names along with their ‘rs’ numbers. To date, we have gathered 7 856 530 known SNP ID/name matches in six species and 5 055 768 SNP ‘rs’ to ‘ss’ ID matches in four species, contributed by 10 research groups, labs and individuals (listed here: https://www.animalgenome.org/tools/SNPnmids/). We have also developed database tools to assist with curator searches or batch matching, allowing copy/paste of an entire data sheet for direct integration of the search results. This tool has greatly accelerated the curation process and has already facilitated the curation of thousands of QTL/association data. These data are available to the public for data integration and annotation, and mining of genome-to-phenome information involving such SNPs.

### Support for multiple genome assemblies within a species for QTLdb

In recent years, there have been more frequent updates of reference genome assemblies (versions) for each species, and more new genome assemblies produced for different breeds or individuals within a species. Unfortunately, due to a lack of resources, NCBI and Ensembl have not been able to annotate all of these alternative assemblies, or there is a delay prior to annotation. Similarly, EVA is not able to expeditiously map all ‘rs’ SNPs to every new assembly.

In order to overcome this barrier, we have developed an in-house pipeline that uses flanking sequences of known SNPs from previously established genome assemblies, to lift (map) them to any new assemblies. Briefly, BEDTools utilities (8) were used to obtain the flanking sequences for known SNPs, and the BWA package (9) was used to map the sequences to a target genome assembly. From trials of about two dozen lift-over experiments, unique mapping results were obtained for 93.4–99.2% of all SNPs, and for SNPs with multiple mappings, 61.8–96.5% were within one chromosome (Supplementary Text Box 1). The same-chromosome mappings were possibly caused by copy number variations. In our implementations, only unique mapping results are retained for QTL/association lift-overs. Supplementary Figure S5 shows five chromosomes as examples to demonstrate linear alignment of coordinate lift-overs from chicken build GG_5.0 to build GRCg7b.

With the SNP mapping tools in place, we were able to develop a QTL/association data lift-over pipeline to distribute curated data to ‘other’ genome builds (note that the tool can be used to place the original data on several different, well-established genome builds; therefore, data lift-over can be multi-directional in order to populate data to all builds). This newly added procedure has greatly accelerated our progress on supporting multiple/new genomes when they become available, shortening wait times from years to only a matter of days.

This method has also effectively enabled curation of custom SNPs represented in QTL/association data before the SNPs are submitted to/released by EVA and have stable ‘rs numbers’. Therefore, we have updated our ‘Minimum information required for Animal QTLdb data entry’ to include SNP probe or flanking sequences, allowing new alternatives for data submission.

### Support for visualization of multiple linked ontologies

**Figure 3.**
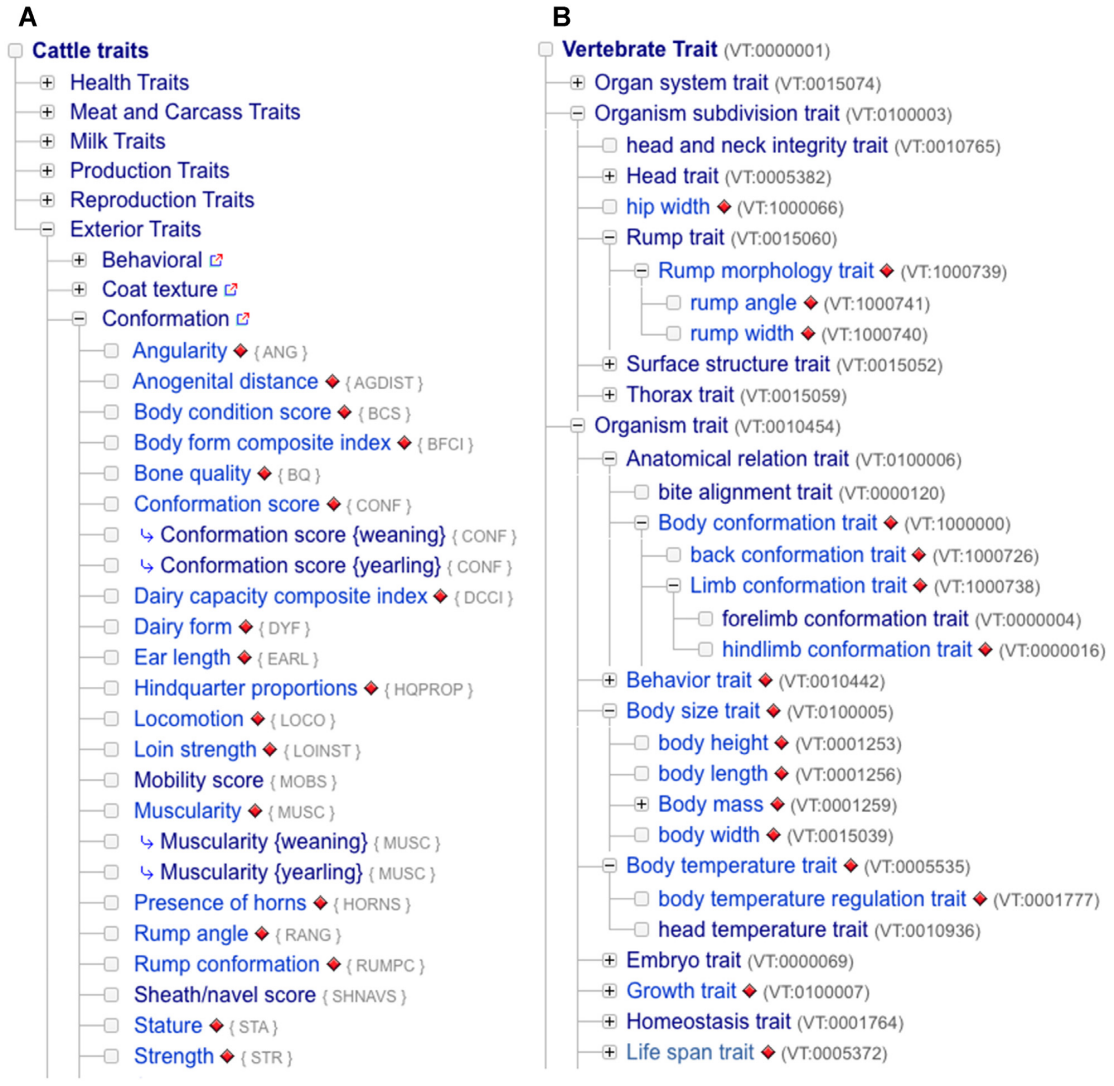
Two trait ontology hierarchy browser views showing the partially expanded QTLdb trait hierarchy (**A**) and Vertebrate Trait Ontology hierarchy (**B**), with QTL/association data links marked where found.

Previously, we have reported integrated development of the Vertebrate Trait (VT), Livestock Product Trait (LPT) and Clinical Measurement (CMO) ontologies and their mapping to traits maintained within QTLdb/CorrDB (6). A new hierarchy display tool has been developed to build an Animal QTLdb web portal for data access using ontology terms from VT/LPT/CMO (Supplementary Figure S6). The display tool also includes search capability, which helps users to quickly find relevant terms in large ontologies. Figure 3 shows two trait ontology hierarchy browser views; each is partially expanded, one for the QTLdb trait hierarchy (A) and one for the Vertebrate Trait Ontology hierarchy (B), with markers denoting the presence of QTL/association data for a particular trait. This function allows database users to peruse the data from different angles and explore new relationships. The tool is available on all species QTLdb front pages and also accessible via the VT, LPT and CMO homepages on the AnimalGenome.ORG website.

### Continued web portal revisions and web interface redevelopment

Web portals to the QTLdb/CorrDB provide platforms that allow integrated access to, and synthesize information from, the backend databases. A good web portal is not just an interface layer to present data but also has built-in functions for integrated and interactive data presentation. We have continually updated our web portals to provide easier and more intuitive data access, via a mixture of browse menus and search options, and functions providing data overviews and expanded views. We design the web pages to work together to formulate various search/browse paths to help users quickly target what they need.

**Figure 4.**
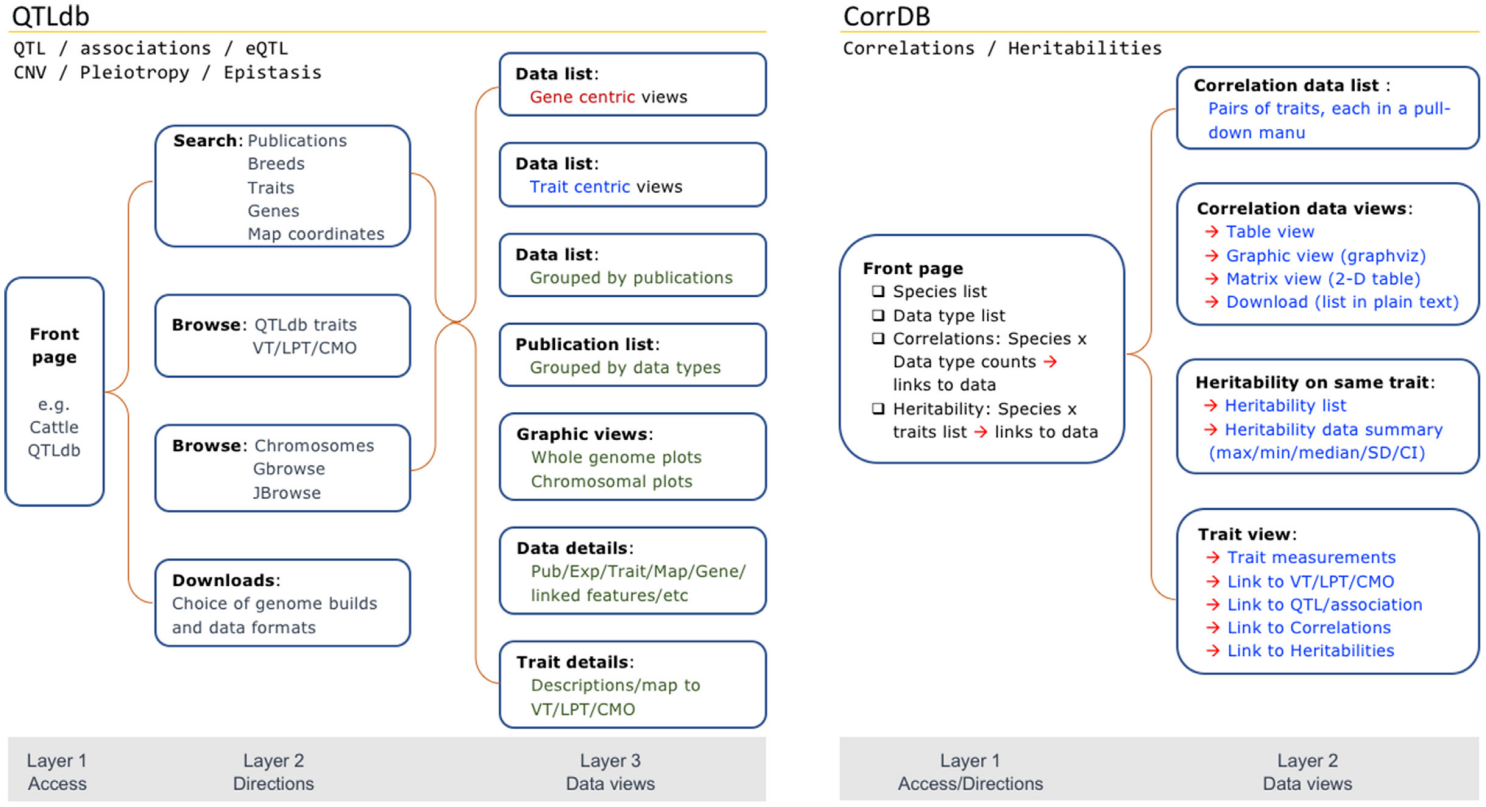
Simplified web portal structure to minimize unnecessary steps for improved access to data while providing tools for various types of data discovery, formatting, and visualization.

As database development has continued over the years and new data types and functions have been added to existing web pages, the underlying web portal trees have been constantly extended. This has made it more complex for users to browse the website. To address this problem, we set out to limit the number of web page layers to under three clicks before a user can reach data. Search options are included on each of the layers. To achieve this, we have carried out extensive reorganization of the web portal structure to improve QTLdb user experience for easier data access by eliminating some intermediate pages for fewer browse/search steps, and to make exploration of the data more intuitive (Figure 4).

On CorrDB, we have placed correlation and heritability data summaries right on the front page (Supplementary Figure S7). On each of the data summary points, hyperlinks are embedded for viewing the data for each species and data type. Correlation data from each link can be presented in Table View, Matrix View, Graphic View, or Download View (page can be saved as text or copied to a blank page). A useful function is to retrieve all data into pull-down lists within a defined size of web page, for manageable and easily explorable access to correlation or heritability data (Supplementary Figure S8). Another helpful new tool is on-the-fly calculation and display of maximum/minimum values, mean, median, standard deviation, interquartile range, and confidence interval of heritability data when multiple measurements exist for the same trait (Supplementary Figure S9).

### Other developments

There have been many ‘small’ but critical tools developed for both QTLdb and CorrDB since our last report (6). In Supplementary Table S1, we summarize 10 functions/tools developed to enhance the database usability.

## DISCUSSION

In addition to Animal QTLdb, a number of databases have incorporated QTL/association data for a variety of species, including human (10,11), rat (12), mouse (13), crops (14–18), model plants (19) and others. While each of these databases has its own unique features and tools, the Animal QTLdb/CorrDB stands out in its continued data growth and development of intuitive web tools not only to access data but also to analyze it on the fly (6). We have also put forth significant effort to ensure that substantial increases in the amount of curated data have not sacrificed data accessibility, in part through the use of intuitive user interfaces and dynamic links between the search outcomes.

Harnessing genomic information including links between genotypes and phenotypes (such as QTL and GWAS data) is critical for livestock improvement in the coming years (20). We believe that the value of a database like QTLdb or CorrDB resides less in how the database is made and how much scientific merit it carries, and more in its utility to a research community or to the public. To this end, efforts are underway to improve the data curation process for QTLdb/CorrDB (not reported here). This includes, but is not limited to, how much and what types of data are included for curation, how data should be structured, and the granularity level of data dissemination. In scaling our efforts, we strive to envision how data may be utilized or mined in the future, and how we can add value to the databases while optimizing the dedication of our time and effort. Many of our efforts in this regard are associated with ontology developments.

From gene mapping to genome sequencing, and from generic linkage map construction to production of individual animal genome assemblies, there has been a shift from QTL discoveries to SNP association studies. This has presented challenges in QTLdb/CorrDB development as we determine the best way to redevelop the database, not only to embrace the future, but also to preserve the value of previously published and curated data (e.g. data preservation considerations between linkage maps and genome maps). To this end, we have taken steps to emphasize back-end data dissections, curation improvements, process fine-tuning, and data management. This groundwork is generally less visible, but provides the basis for changes in the future. Although we have started to proceed with QTLdb migration from linkage map to genome map based data support, it may take some time to complete the transition, because the data availability and research community needs are different for each of the species. During the process, we may need to accommodate both map types.

After >20 years of QTL/association studies in livestock animals, genotype/phenotype association data are still accumulating at an increasing rate. It is our hope that the continued effort toward building and improving the QTLdb and CorrDB will expand the possibilities to harness these big data to better comprehend the relationships between livestock genotypes and phenotypes.

## DATA AVAILABILITY

QTLdb: https://www.animalgenome.org/QTLdb/; CorrDB: https://www.animalgenome.org/CorrDB/. In addition, the data is also available upon each release at several data alliance partner websites, including NCBI: http://www.ncbi.nlm.nih.gov/gene; Ensembl: http://www.ensembl.org/; UCSC: https://genome.ucsc.edu/cgi-bin/hgGateway; Reuters Data Citation Index: http://wokinfo.com/products_tools/multidisciplinary/dci/.

## Supplementary Material

gkab1116_Supplemental_Files
