# Supplementary material for: Bringing the Animal QTLdb and CorrDB into the future: meeting new challenges and providing updated services
Source: Nucleic Acids Res. 2021 Nov 24;50(D1):D956–61. doi: 10.1093/nar/gkab1116 (PMC8728226; doi:10.1093/nar/gkab1116)

**Supplementary Figure 1s**

Starting point to initiate instances for species/genome builds information


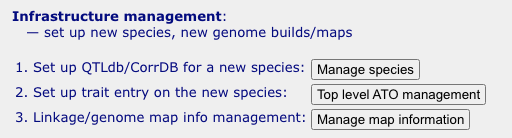


**Supplementary Figure 2s**

Web form to manage species within QTLdb/CorrDB


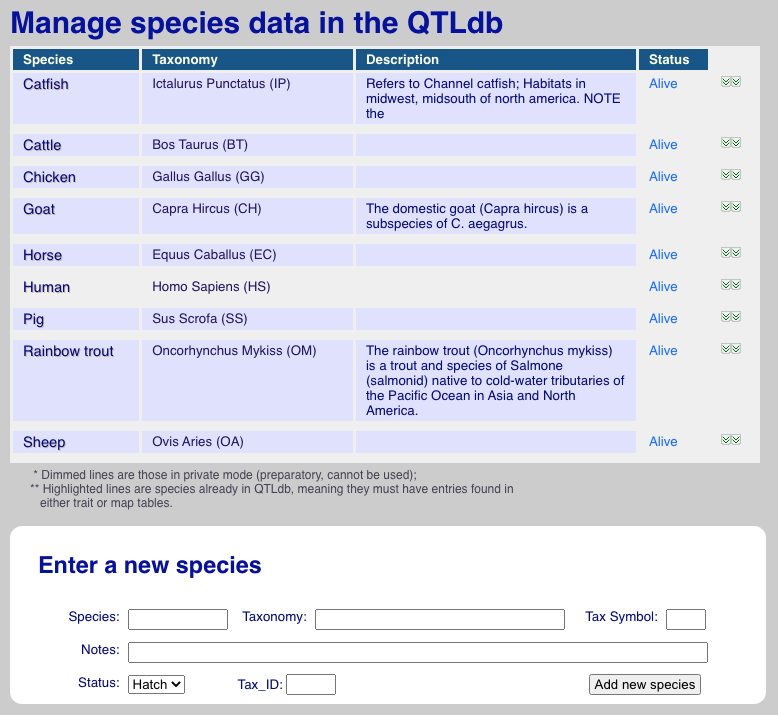


**Supplementary Figure 3s**

Web form to set up database structure for trait entries for a newly added species (the curators/ editors will be able to enter new or modify existing trait information after this set up).


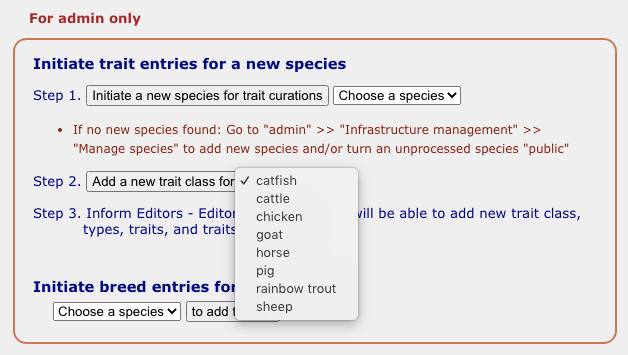


**Supplementary Figure 4s**

Web form to add/manage/review genome build information


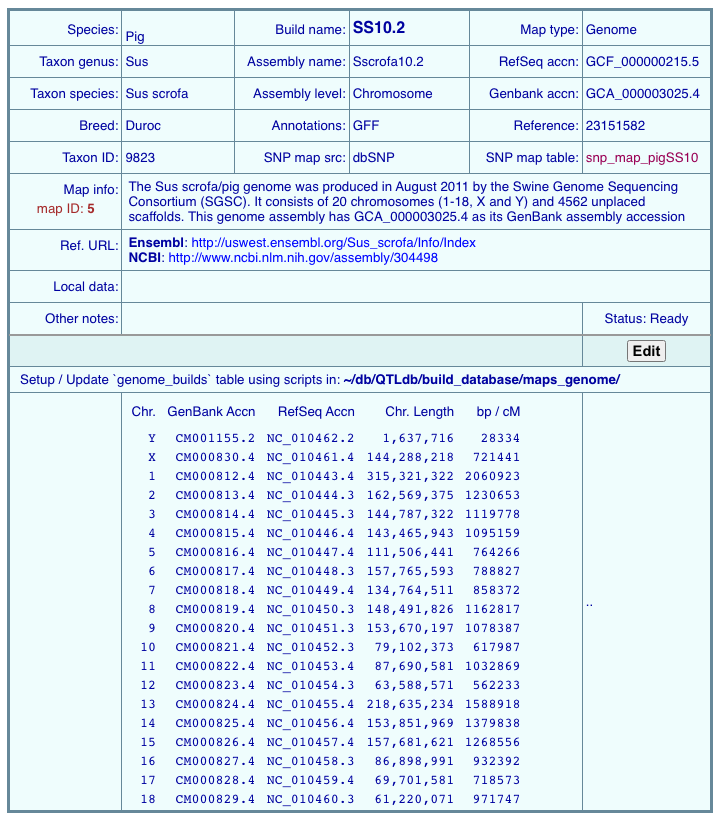


**Supplementary Figure 5s**

Alignments of SNP lift-over coordinates between chicken builds GG_5.0 and GRCg7b to demonstrate the concordance of the lift-over maps (example data is shown for chromosomes 1-5).

**
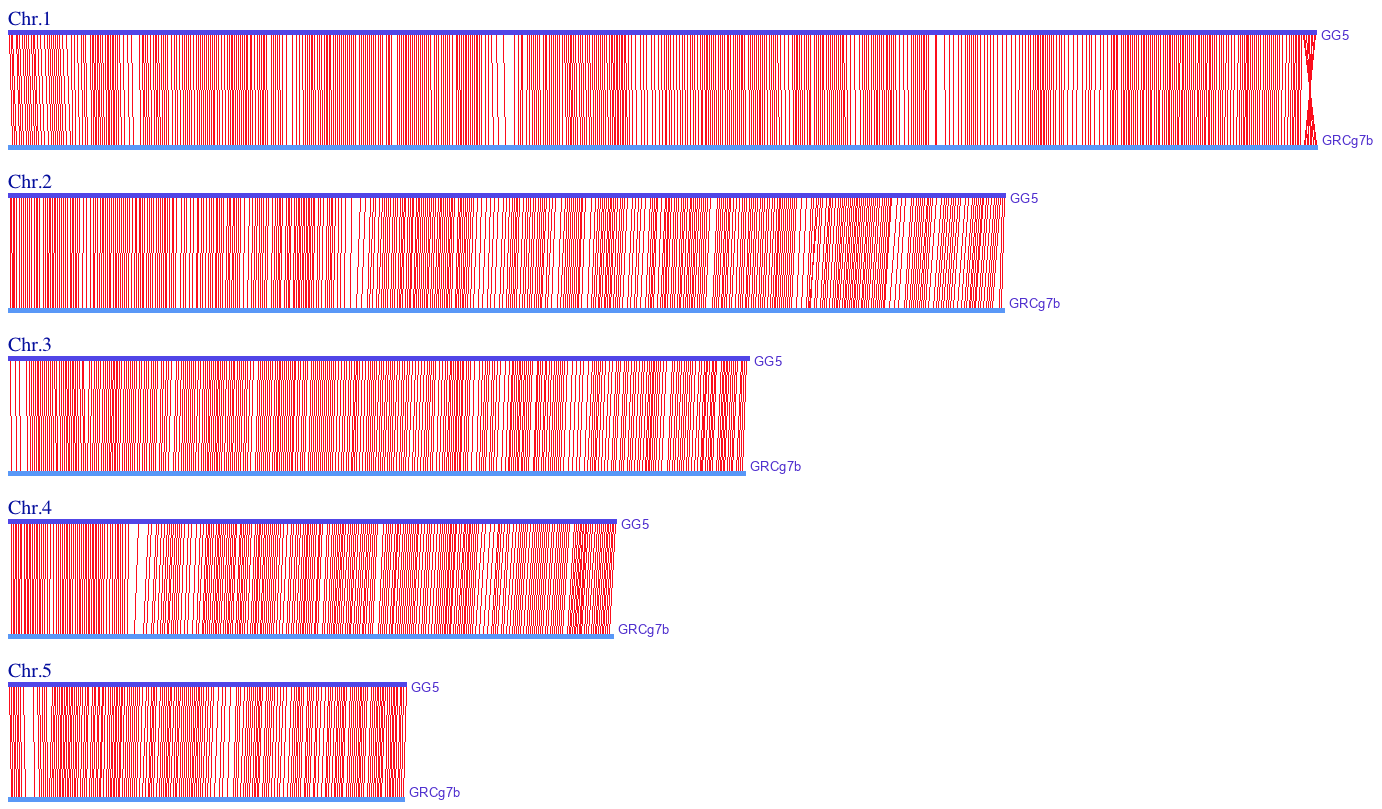
**

**Supplementary Figure 6s**

A view of a webpage displaying options for accessing QTLdb data by different trait ontologies via search or browse.


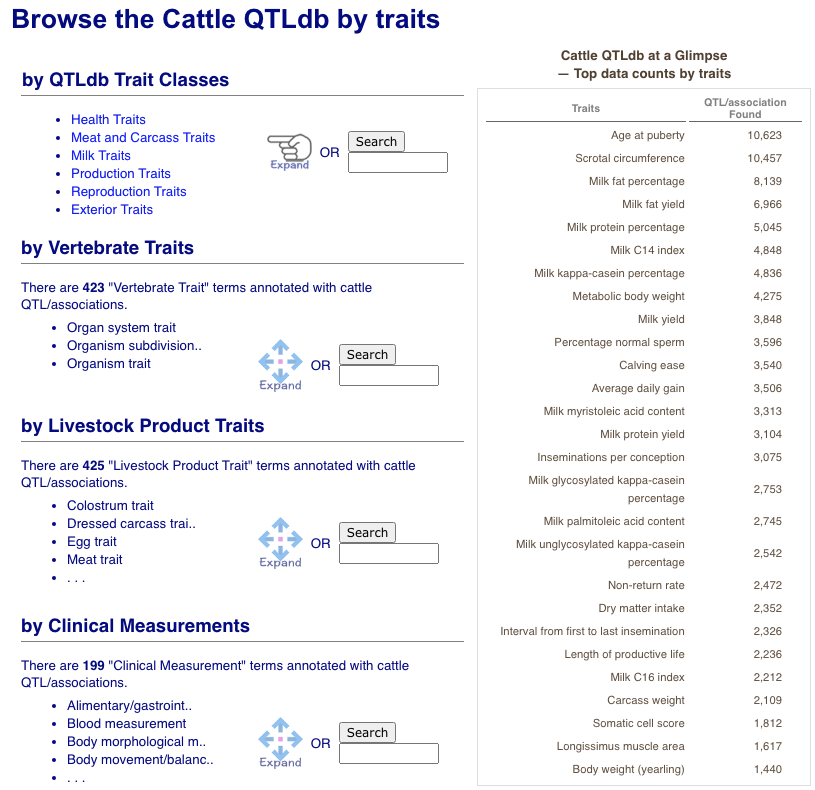


**Supplementary Figure 7s**

Easy access to correlation data is provided with pull-down lists of all traits on one page. This saves search time because users can more easily see what data is available.


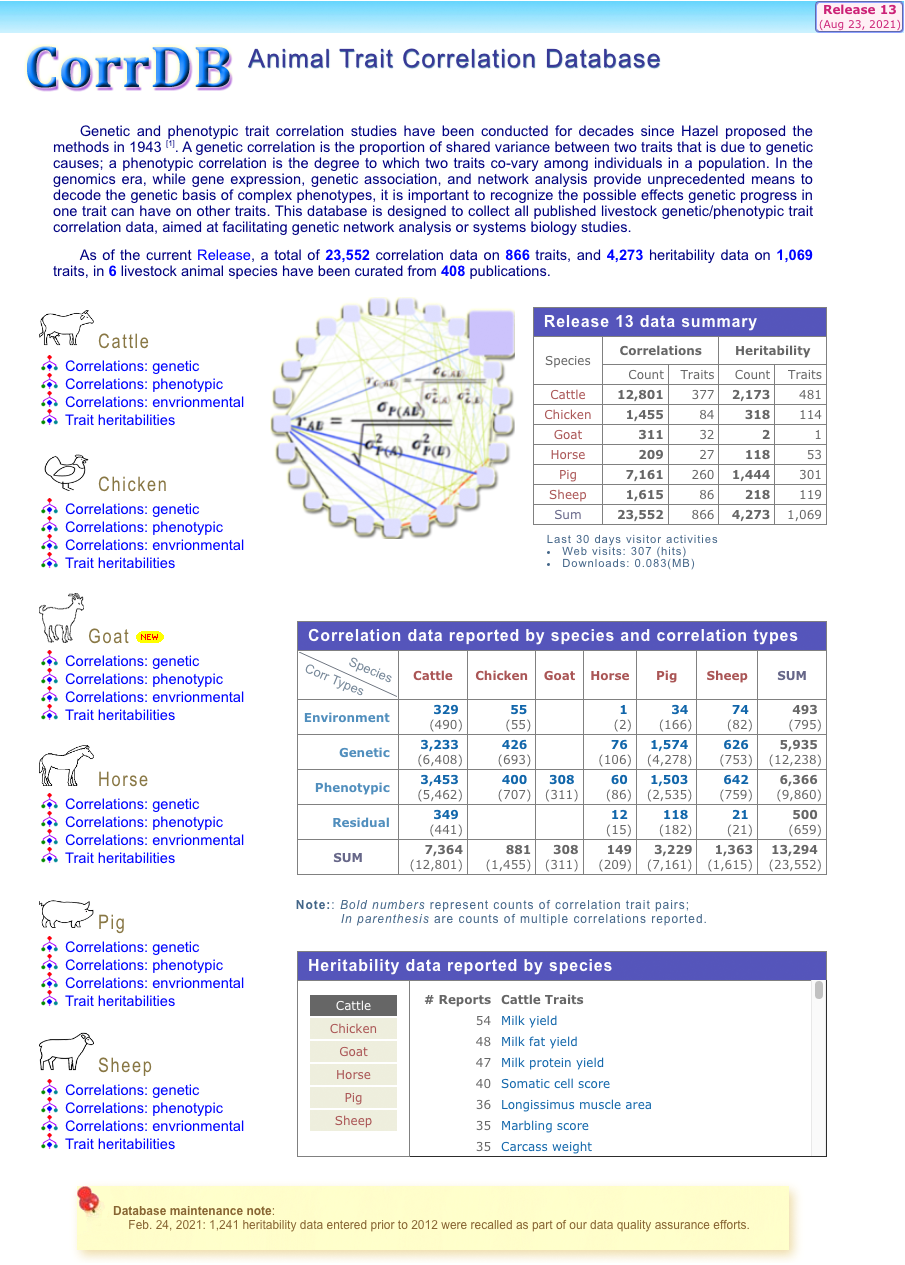


**Supplementary Figure 8s**

Easy data access to correlation data is provided with pull-down lists of all traits on one page. This effectively saves time for users from guessing what to search for.


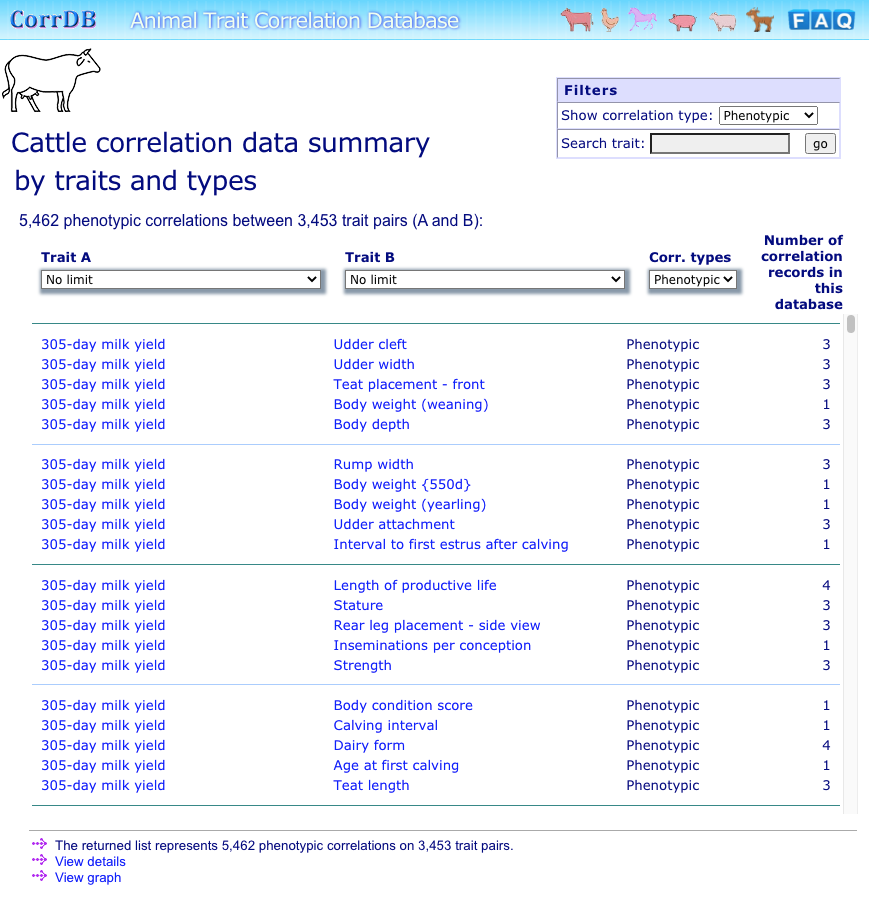


**Supplementary Figure 9s**

A data summary panel for traits having multiple heritability measurements. In the example, the median, interquartile range, maximum/minimum, mean, and 95% confidence interval are calculated on the fly using embedded R functions.


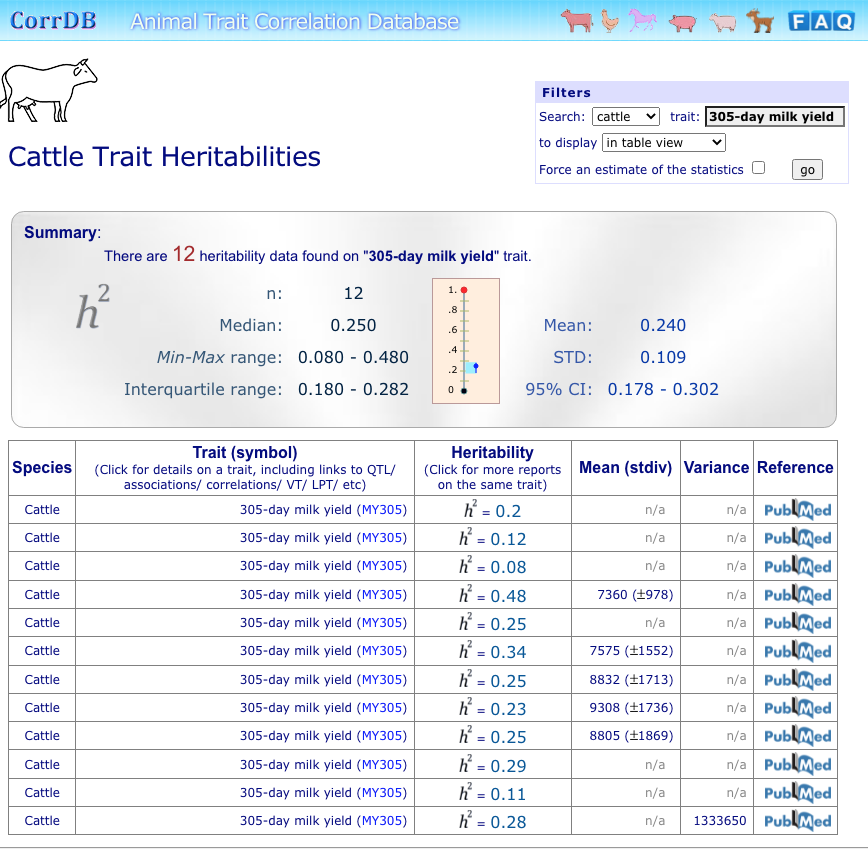

Supplement: gkab1116_Supplemental_Files [file gkab1116_supplemental_files.zip › Supplementary_Figures_1s-9s.docx]
