# Supplementary material for: Bringing the Animal QTLdb and CorrDB into the future: meeting new challenges and providing updated services
Source: Nucleic Acids Res. 2021 Nov 24;50(D1):D956–61. doi: 10.1093/nar/gkab1116 (PMC8728226; doi:10.1093/nar/gkab1116)
Supplement: gkab1116_Supplemental_Files [file gkab1116_supplemental_files.zip › Supplementary_Table_1s.docx]

Supplementary Table 1s.

Some recent database utility developments.

|  | Utility | Description |
| --- | --- | --- |
| 1 | DBxref for external data links | New dbxref data link services were added to both Animal QTLdb and CorrDB to allow external URL links to specific data in the databases (across species). Such links are often used by web services, API tools, or database xref pointers. The syntax for QTLdb: https://www.animalgenome.org/QTLdb/q?id=[ID]  The syntax for CorrDB: https://www.animalgenome.org/CorrDB/q?id=[ID]  (Replace "ID" with the unique numerical identifier for a specific data point.)  In addition, the QTLdb and CorrDB are both registered at *Identifiers.org,* a unique site for universal database and data links. Search for “QTLdb” and “CorrDB” in the site’s registry. |
| 2 | Visibility of eQTL data | Curated gene-eQTL data can now be delivered through web tools in the Animal QTLdb ("trait-eQTL" have been available since 2009). The web links include lists by publication through permanent record locator, QTL/association data details, and a number of search results pages. |
| 3 | SNP lookup tool based on genome coordinates | A data curation helper tool has been developed to facilitate flanking SNP lookup for given genome intervals. The tool picks the leftmost and the rightmost SNPs as flanking candidates for a searched span, along with their coordinates for curators to determine the use of the queried SNPs for data annotations. The SNP query link has been added to "trait search", "gene search", "location search", and "general search" tools. |
| 4 | DOI (Digital Object Identifier) | DOI (Digital Object Identifier) data in the Animal QTLdb is now visible on the abstract view and data summary view pages. Users can follow these links to the full text of a publication when they are available. |
| 5 | Visibility of epistasis and pleiotropy data | Epistasis and pleiotropy data were introduced for curation into the Animal QTLdb in 2009 and 2014, respectively. New implementations were made to allow these data to be viewed in the public portal (e.g., in the data summary for publications; epistatic data display; pleiotropic data display). |
| 6 | Batch upload tool for curators | A new batch upload tool was integrated into the Animal QTLdb curator/editor tools for external curators to upload pre-formatted data prepared according to the Minimum Required Information Guidelines. An uploaded data processing pipeline was developed to allow proper data flow and routing of jobs between external curators, QTLdb curators, QTLdb editors, and database administrators. |
| 7 | Links between related studies (references) | Some curated QTL/association reports in the Animal QTLdb can be related because they are follow-up studies, combined analyses of previous experiments, reanalysis, confirmation studies, or multiple reports from the same experiments. Now this information is collected in the QTLdb and can be seen on publication information pages, for example, the abstract page (e.g., https://www.animalgenome.org/cgi-bin/QTLdb/BT/qabstract?PUBMED_ID=30644110) or curated data summary page (when viewing list of QTL/associations from a publication, e.g., https://www.animalgenome.org/QTLdb/supp/?t=RbEq9K9LtC). |
| 8 | Dynamic data download | Dynamic data download options have been added to the Animal QTLdb "Trait Centric" and "Gene Centric" views (accessible through "Search and Analysis" tools), for which all data shown on a search results page can be downloaded. In this way, users can relatively easily locate data of interest and download them for further data mining/analysis. |
| 9 | Curator tool usage aid | On-site usage guides/alerts have been added where certain operations can be triggered in the curation pipeline. This aid helps the curators to proceed intuitively with curation, reducing the time/need to read and understand Curation Manuals. |
| 10 | Collaborative curation work flow | Different curators/editors can work together by sharing their intermediate steps in the curation data flow pipeline. |
