# Supplementary material for: Bringing the Animal QTLdb and CorrDB into the future: meeting new challenges and providing updated services
Source: Nucleic Acids Res. 2021 Nov 24;50(D1):D956–61. doi: 10.1093/nar/gkab1116 (PMC8728226; doi:10.1093/nar/gkab1116)
Supplement: gkab1116_Supplemental_Files [file gkab1116_supplemental_files.zip › Supplementary_Text_Box_1.docx]

**Supplementary data box**:

A summary of quality control data on the SNP coordinates liftover process (taken from direct outputs of scripts running the process).

Coordinates Liftover: A generic data re-mapping pipeline using SNPs as an example.

A Summary

1. Steps and time clapsed at each step ( 8 hrs 24 min):

____Pig_____ ___Chicken____ ____Sheep_______

0) Old genome samtool indexing ................... 1 min ........ 1 min .......... 1 min

New genome bwa indexing ...................... 47 min ....... 18 min ......... 58 min

1) Query db for SNP coordinates .................. 2 min ........ 1 min .......... 2 min

2) Obtain flank seqs from old genome ............. 5 min ........ 2 min .......... 4 min

3) Convert flank seqs from fasta to fastq ....... 11 min ........ 4 min ....

Mapping fastq seqs to new genome ....... 6 hrs 43 min .. 1 hr 14 min .. 18 hrs 15 min

4) Parse results, replace IDs .................... 7 min ........ 3 min .......... 7 min

5) Process results to eliminate duplicates ....... 3 min ........ 2 min .......... 5 min

6) load into mysql db ........................... 25 min ........ 6 min ......... 12 min

8 hrs 24 min 1 hrs 37 min 19 hrs 44 min

Genome size (bps)........... 2,425,476,477 .. 939,467,996 .. 2,869,914,396

Ave. chr.size (bps/chr)....... 127,656,656 ... 49,445,684 .... 110,381,322

Num. of SNPs .................. 65,144,393 ... 22,765,359 ..... 52,985,452

Num. of chromosomes ................... 19 ........... 29 ............. 26

2. Data summary: number of SNPs with multiple map locations:

(1) Pig SS_11 to SS_MARC1:

Multiple

locations Number of SNPs

1 61,300,525

2 1,694,106

3 192,898

4 51,390

5 11,947

6 7,008

7 1,560

8 955

9 412

10 167

11 29

12 88

13 6

14 13

15 5

16 7

18 5

20 1

21 1

27 1

Dups: 4,300,167

Uniq: 61,300,525

Total: 65,600,692

Unique rate: 93.4449%

(2) Chicken GG_5 to GRCg7b:

Counting the number of SNPs with multiple map locations (regardless which chr) ...

Multiple locations Number of SNPs

1 21,741,262 97.605504%

2 497,976 2.235620%

3 31,164 0.139908%

4 3,823 0.017163%

5 209 0.000938%

6 185 0.000831%

7 1 0.000004%

9 6 0.000027%

10 1 0.000004%

3. Saving data

Saving unique mapping results into "snp_map_chickenGG5.to.GRCg7b.dat"...

Multi mapping: 533,365 (ignored)

Uniq mapping: 21,741,262 (saved)

Total: 22,274,627

Unique rate: 97.61%

Of all multiple mapping results, how many are mapped to different Chrs

Number of Number of Proportion

Different SNPs in this of multi-Chr

Chromosomes MultiChr Grp mappings

----------- ------------ ------------

1 514,834 96.526% (likely copy number variations)

2 17,992 3.373% (the rest: likely erors)

3 522 0.098%

4 17 0.003%

Sum 533,365
